# Identification and Characterization of High-Molecular-Weight Glutenin Subunits from *Agropyron intermedium*

**DOI:** 10.1371/journal.pone.0087477

**Published:** 2014-02-04

**Authors:** Shuanghe Cao, Zhixin Li, Caiyan Gong, Hong Xu, Ran Yang, Shanting Hao, Xianping Wang, Daowen Wang, Xiangqi Zhang

**Affiliations:** 1 State Key Laboratory of Plant Cell and Chromosome Engineering, Institute of Genetics and Developmental Biology, Chinese Academy of Sciences, Beijing, China; 2 College of Agriculture, Yangtze University, Jingzhou, Hubei, China; 3 College of life sciences, Northwest Sci-Tech University of Agriculture and Forestry, Yangling, Shanxi, China; Agriculture and Agri-Food Canada, Canada

## Abstract

High-molecular-weight glutenin subunit (HMW-GS) is a primary determinant of processing quality of wheat. Considerable progress has been made in understanding the structure, function and genetic regulation of HMW-GS in wheat and some of its related species, but less is known about their orthologs in *Agropyron intermedium*, a useful related species for wheat improvement. Here seven HMW-GSs in *Ag. intermedium* were identified using SDS-PAGE and Western blotting experiments. Subsequently, the seven genes (*Glu-1Aix1*∼*4* and *Glu-1Aiy1*∼*3*) encoding the seven HMW-GSs were isolated using PCR technique with degenerate primers, and confirmed by bacterial expression and Western blotting. Sequence analysis indicated that the seven *Ag. intermedium* HMW-GSs shared high similarity in primary structure to those of wheat, but four of the seven subunits were unusually small compared to the representatives of HMW-GS from wheat and two of them possessed extra cysteine residues. The alignment and clustering analysis of deduced amino acid sequences revealed that 1Aix1 and 1Aiy1 subunits had special molecular structure, belonging to the hybrid type compounding between typical x- and y-type subunit. The xy-type subunit 1Aix1 is composed of the N-terminal of x-type and C-terminal of y-type, whereas yx-type subunit 1Aiy1 comprises the N-terminal of y-type and C-terminal of x-type. This result strongly supported the hypothesis of unequal crossover mechanism that might generate the novel coding sequence for the hybrid type of HMW-GSs. In addition to the aforementioned, the other novel characteristics of the seven subunits were also discussed. Finally, phylogenetic analysis based on HMW-GS genes was carried out and provided new insights into the evolutionary biology of *Ag. intermedium*.

## Introduction

In common wheat (*Triticum aestivum* L.) and related species, high-molecular-weight glutenin subunit (HMW-GS) is an important group of seed storage proteins and shares the primary structure that includes the conserved signal peptide (which is excised from the mature HMW-GS), N- and C-terminal domains, and the central repetitive region composed of tri-, hexa- and nonapeptide motifs [1], [2], [3]. Mainly judging from the sizes and sequences of conserved N-terminal domains (especially, the number and location of cysteine residues), HMW-GSs are categorized into x- and y-type encoded by *Glu-1-1* and *Glu-1-2* in the *Glu-1* locus, respectively [1], [4]. In addition to wheat, many orthologous HMW-GSs have been identified and isolated from related species (reviewed in [5]). Compared to wheat, quite a few HMW-GSs from related species possess an unusual number and/or location of cysteine residues, which are involved in the formation of inter- or intra-molecular disulphide bonds, and accordingly have an important effect on the network of gluten macro polymer (GMP) [6], [7], [8]. Noticeably, two hybrid HMW-GSs were identified from *Aegilops searsii* and *Triticum aestivum-Agropyron intermedium* disomic alien addition lines, respectively [9], [10]. Owing to their role in determining the strength and elastic properties of the gluten complex, and their conservation in wheat and related species [1], [3], [7], continued mining for novel HMW-GSs from wild germplasm may be beneficial for not only improving wheat end use quality but also studying the structural variation and evolution of this important protein family.

*Ag. intermedium* (Host) Beauvoir ( = *Thinopyrum intermedium* (Host) Barkwarth = *Elytrigia intermedia* (Host) Nevski, 2n = 6x = 42), being one of the wild relatives of wheat, possesses many desirable characteristics for wheat improvement, such as high grain protein content, resistance to many diseases caused by fungi or viruses, tolerance to low temperature, drought, moisture and salt stresses, perennial habits and high crossability with wheat, etc (reviewed in [11]). Because of its high crossability with wheat, a number of useful genes have been transferred from this species to common wheat through chromosome engineering, which has led to the development of many useful wheat germplasm, including partial amphiploids, addition, substitution and translocation lines [12], [13], [14], [15], [16]. *Ag. intermedium* has been one of the most important related species. Sun (1981) developed five partial amphiploids of *T. aestivum-Ag. intermedium*, i.e., octoploid *Trititrigia* Zhong1∼5 (2n = 8x = 56) through crossing common wheat with *Ag. intermedium* [15]. Furthermore, He et al. (1988) developed two sets of *T. aestivum-Ag. intermedium* disomic alien addition lines (TAI-I & TAI-II series) based on Zhong 1∼5 [16]. We measured the quality parameters of TAI series lines and found that some of them have good quality (data not shown). In addition, several high-quality varieties were bred and released in China, such as Shanmai150, Gaoyou503, Xiaobingmai33 and so on, using these octoploid *Trititrigia* or their derived lines. Thus, it is valuable to identify and characterize *Ag. intermedium* glutenin subunits for improving the processing property of wheat flour, considering that its quality is largely determined by the composition of glutenin subunits [1], [3], [7].

We previously identified and characterized a novel *Ag. intermedium* HMW-GS gene (*Glu-1Aix1*) from a *T. aestivum-Ag. intermedium* addition line TAI-13. In addition to belonging to the hybrid type mentioned above, the subunit 1Aix1 possesses two extra cysteine residues in the repetitive region [10]. In the present study, we identified and characterized a series of HMW-GSs and their coding sequences from *Ag. intermedium* and further investigated their evolutionary biology with phylogenetic analysis.

## Materials and Methods

### Plant Materials

The *Ag. intermedium* line in this study was kindly provided by Professor Mengyuan He (Northeast Normal University, Changchun, China) and its accession number is No. 33826 in N. I. Vavilov Research Institute of Plant Industry, Russia. This *Ag. intermdium* line has been utilized to create the chromosomal engineering lines, Zhong 1∼5 and TAI series, which are good “bridge” parents of wheat breeding [15], [16]. Common wheat variety Chinese Spring was used as a standard for assessing the electrophoretic mobilities of the HMW-GSs from *Ag. intermedium*.

### Glutenin Preparation, SDS-PAGE and Western Blotting

The glutenin proteins were extracted from mature seeds of *Ag. intermedium* and Chinese Spring according to the method described by Fu and Kovacs [17] and were separated by SDS-PAGE. The electrophoresis was carried out at 160 volts for 1.5 hour using 12% gel of Laemmli basic discontinuous system in Mini-PROTEAN® 3 Cell (BIO-RAD), and the proteins were stained with Coomassie Brilliant Blue R-250 [18]. The HMW-GSs were identified and confirmed by Western blotting analysis according to the method described by Liu et al. [19].

### DNA Extraction and PCR

Genomic DNA was extracted from the young leaves of *Ag. intermedium* with CTAB method [20]. For amplifying HMW-GS genes of *Ag. intermedium* by genomic PCR, a pair of degenerate primers, P1 (5′-ATGGCTAAGCGGC/TTA/GGTCCTCTTTG-3′) and P2 (5′-CTATCACTGGCTG/AGCCGACAATGCG-3′), was designed according to published DNA sequences of wheat HMW-GS genes [21]. The primer P1 contained the start codon of the HMW-GS gene’s ORF (open reading frame), and the primer P2 possessed the two tandem stop codons that are conserved almost in all of HMW-GS genes characterized so far. Genomic PCR was carried out using high fidelity *LA Taq* polymerase with GC buffer (Takara). The parameters for the PCR reaction were one step at 94°C for 5 min, followed by 35 cycles of 94°C for 1 min, 65°C for 40 sec and 72°C for 3 min, and a final extension step at 72°C for 7 min.

### Isolation and Confirmation of Complete ORFs

PCR products were separated in 1% agarose gels. DNA fragments of expected sizes were recovered from agarose gels, and then were ligated into the pGEM-T vector (Promega). The competent cells of *Escherichia coli* DH10B were transformed with the ligation reactions following the standard procedures (Promega). The positive clones were selected with blue/white color screening and sequenced by a commercial company (Bioasia Biotechnology Company). The sequencing of HMW-GS gene is stumbled by tandem and interspersed repeats in its central region. In order to obtain the complete ORFs, their nest-deleted subclones were prepared according to the protocol: the purified plasmid of positive clone was digested by *Nco*I, *Apa*I, Exonuclease III and S1 Nuclease in turn, and then their products self-looped by T4 DNA ligase were transformed into competent cells of *E. coli* DH10B. The identification and sequencing of the subclones were carried out as described above. The complete ORF sequences were determined through overlapping subclones created by the nested deletion method.

### Bacterial Expression and Western Blotting Analysis

For bacterial expression of the mature HMW-GSs from *Ag. intermedium*, a series of PCR primers (Table 1) was designed to amplify mutant ORFs from which the coding sequences of signal peptides were removed, and appropriate restriction enzyme sites (*Nde*I and *Eco*RI) were introduced to facilitate subsequent cloning experiments. PCR parameters for amplifying the mutant ORF are identical with the described above. After cloning the mutant ORFs into the expression vector pET-30a (Invitrogen), positive constructs were selected for expressing the mature proteins of the target HMW-GSs in bacterial cells. The conditions for inducing bacterial expression of pET construct are same as detailed by Wan et al. [22]. *E. coli* BL21 (DE3) pLysS cells were transformed with the pET plasmids containing the HMW-GS genes. The positive clones were cultured in LB liquid medium containing 35 µg/mL kanamycin in a shaking incubator at 37°C until the OD_600_ reached 0.6, and then the expression of target HMW-GSs were induced by adding 1 mM IPTG into the medium. After incubation overnight, the *E. coli* cells were collected by centrifugation at 5,000 g and 4°C for 10 min, and the expressed proteins were subsequently extracted using 50% (v/v) 1-propanol containing 2% (w/v) DTT. To confirm the identity of the bacterially expressed proteins directed by pET constructs, Western blotting experiments using the polyclonal antibody specific for HMW-GSs were conducted [19].

**Table 1:** PCR primers used in bacterial expression experiments.

Primer pair	Sequence^a^	Restriction site introduced	Mutant ORF amplified
E1	5′ -ACCCATATGGAAGGTGAGGCCTCTG-3′	*Nde*I	1Ai4
	5′ -CTAGAATTCCTATCACTGGCTGGCC-3′	*Eco*RI	
E2	5′ -ACCCATATGGAAGGTGAGGCCTCTG-3′	*Nde*I	1Ai1
	5′ -CTAGAATTCCTATCACTGGCTAGCC-3′	*Eco*RI	
E3	5′ -ACCCATATGGAAGGTGAGGCCTCTG-3′	*Nde*I	1Ai5
	5′ -CTAGAATTCCTATCACTGGCTGGCC-3′	*Eco*RI	
E4	5′ -ACCCATATGGAAGGTGAGGCCTCTG-3′	*Nde*I	1Ai6
	5′ -CTAGAATTCCTATCACTGGCTAGCC-3′	*Eco*RI	
E5	5′ -ACCCATATGGAAGGTGAGGCCTCTA-3′	*Nde*I	1Ai2
	5′ -CTAGAATTCCTATCACTGGCTGGCC-3′	*Eco*RI	
E6	5′ -ACCCATATGGAAGGTGAGGCCTCTA-3′	*Nde*I	1Ai3
	5′ -CTAGAATTCCTATCACTGGCTGGCC-3′	*Eco*RI	
E7	5′ -ACCCATATGGAAGGTGAGGCCTCTA-3′	*Nde*I	1Ai7
	5′ -CTAGAATTCCTATCACTGGCTGGCC-3′	*Eco*RI	

a The underlined nucleotides constitute the restriction sites listed in the third column.

### Comparison of Deduced Amino Acid Sequences and Clustering Analysis

The amino acid sequences were deduced from the HMW-GS genes of *Ag. intermedium* by ORF Finder program (http://www.ncbi.nlm.nih.gov/gorf/gorf.html) and compared to previously published HMW-GSs through multiple alignments using DNAMAN 5.2.2 (Lynnon BioSoft, http://www.lynnon.com/). Homology tree with observed divergency was used for clustering analysis based on the multiple alignments. Some of representative HMW-GSs from *Triticum* genus, 1Ax2* (M22208), 1Bx7 (X13927), 1Dx5 (X12928), 1Ay (AJ306977, from *T. timopheevi*), 1By9 (X61026) and 1Dy10 (X12929), were selected for comparing to those of the *Ag. intermedium*.

### MALDI-TOF-MS

The seed protein samples prepared from *Ag. intermedium* were separated using 10% SDS-PAGE with the Tris-Glycine-SDS running buffer. After Coomassie Brilliant Blue R-250 staining, the native target subunits were manually excised from the gel, followed by in gel digestion with trypsin [23]. The samples of the digested protein were analyzed in an Autoflex MALDI-TOF-MS (Matrix-Assisted Laser Desorption/Ionization Time of Flight Mass Spectrometry) (Bruker Daltonics) with a mass range of m/z 1000 to 15000 Da. The MALDI-TOF-MS measured peptide mass spectra were compared to the calculated mass spectra, which were predicted based on the trypsin-digested amino acid sequence deduced from the target gene using the bioinformatic program PeptideMass (http://web.expasy.org/peptide_mass/). The MALDI-TOF-MS analysis was repeated three times using the protein samples prepared from separate batches of seeds.

### Phylogenetic Analysis

To analyze the phylogenetic relationship between *Ag. intermedium* and the other species in *Triticeae*, HMW-GS sequences of representative species in the tribe were collected from GenBank (http://www.ncbi.nlm.nih.gov/genbank/). For calculating evolutionary distances and constructing phylogenetic trees, Clustal W program [24] and the MEGA program (Version 5.2, http://www.megasoftware.net/) were used.

## Results

### Identification of HMW-GSs in *Ag. intermedium*

**Figure 1:**
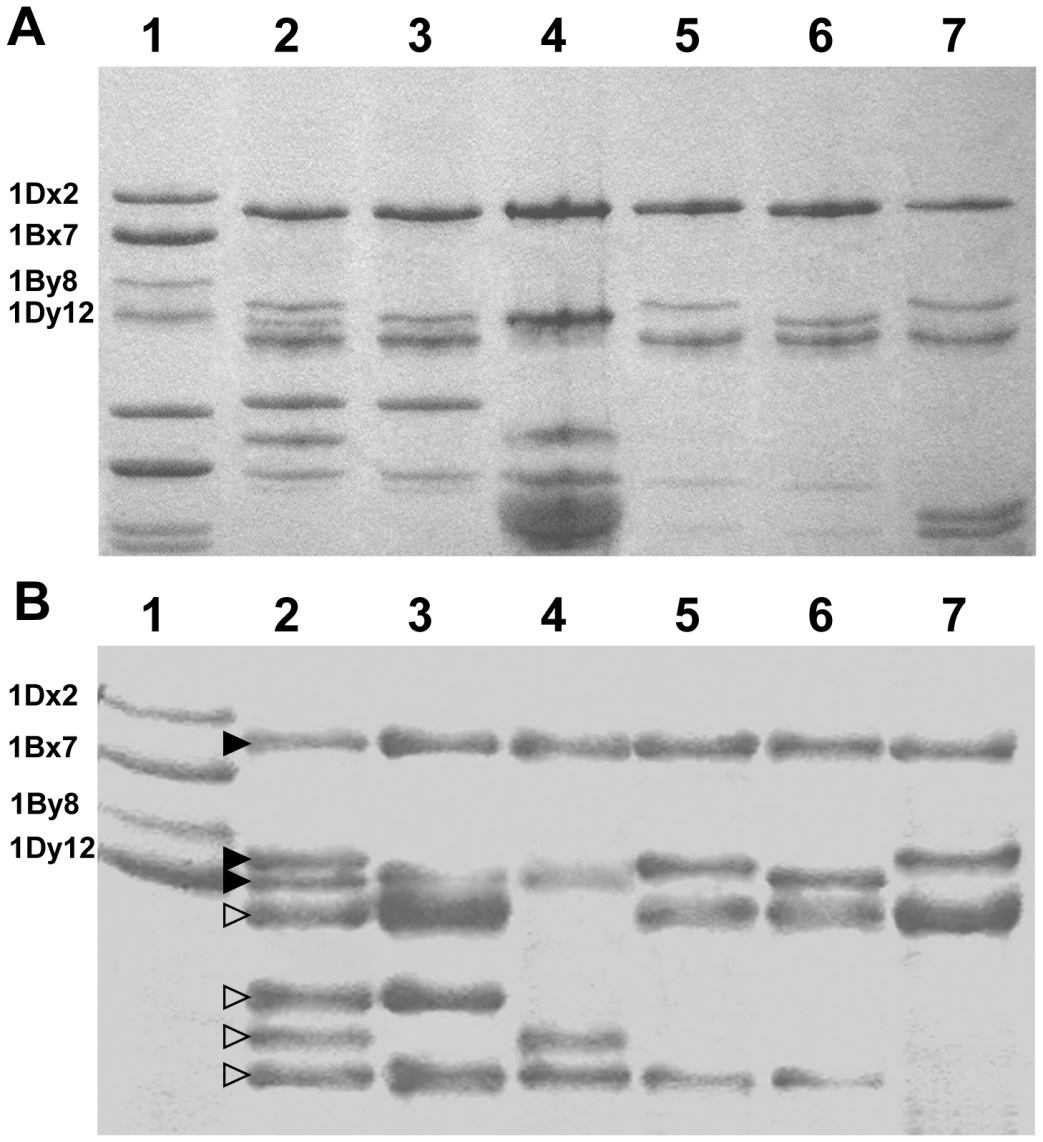
SDS-PAGE (A) and Western blotting (B) analysis of HMW-GSs of *Ag. intermedium*. Lane 1 shows the named HMW-GSs from common wheat variety Chinese Spring as a control. Lanes 2∼7 show the HMW-GSs from six representative seeds of the *Ag. intermedium* line used in this study. The seven expressed HMW-GSs with distinct electrophoretic mobility comparing with Chinese Spring were detected by SDS-PAGE (A) and were confirmed using Western blotting experiment with polyclonal antibody specific for HMW-GSs (B). Among the seven HMW-GSs from *Ag. intermedium*, three subunits (marked with solid triangles in lane 2 of B) share comparable electrophoretic mobility with Chinese Spring, the other four subunits (marked with hollow triangles in lane 2 of B) moved faster than those HMW-GSs from Chinese Spring.

SDS-PAGE protein profiles of individual seeds of *Ag. intermedium* showed that quite a few protein bands were distributed in HMW-GS region of common wheat variety Chinese Spring (lanes 2∼7 in Figure 1A). For identifying HMW-GSs, Western blotting experiment with the polyclonal antibody specific for HMW-GS was carried out. The Western blotting patterns indicated that most of the seeds used in this study expressed 3 to 5 HMW-GSs (lanes 3∼7 in Figure 1B), but only one possessed seven HMW-GSs (lane 2 in Figure 1B). By comparing the bands detected by Western blotting, seven unique HMW-GSs with distinct electrophoretic mobilities were identified in the *Ag. intermedium* total seed protein extracts (more than 100 seeds tested). This means that the seven HMW-GSs expressed in the seed shown in lane 2 of Figure 1 covered those from other seeds. Thus, they probably represent all of the HMW-GSs from the three genomes of the *Ag. intermedium* line (E1E2X, 2n = 6x = 42) used in this study [25] and were tentatively designated as 1Ai1, 1Ai2, 1Ai3, 1Ai4 1Ai5, 1Ai6 and 1Ai7, respectively (from higher to lower molecular mass in lane 2 in Figure 1). In term of their electrophoretic mobilities, three of the seven subunits (1Ai1∼3, marked with the solid triangles in Figure 1B) were comparable to the HMW-GSs from Chinese Spring, and the others (1Ai4∼7, marked with hollow triangles in lane 2 of Figure 1B) were unusually small.

### Isolation of HMW-GS Genes from *Ag. intermedium*

**Figure 2:**
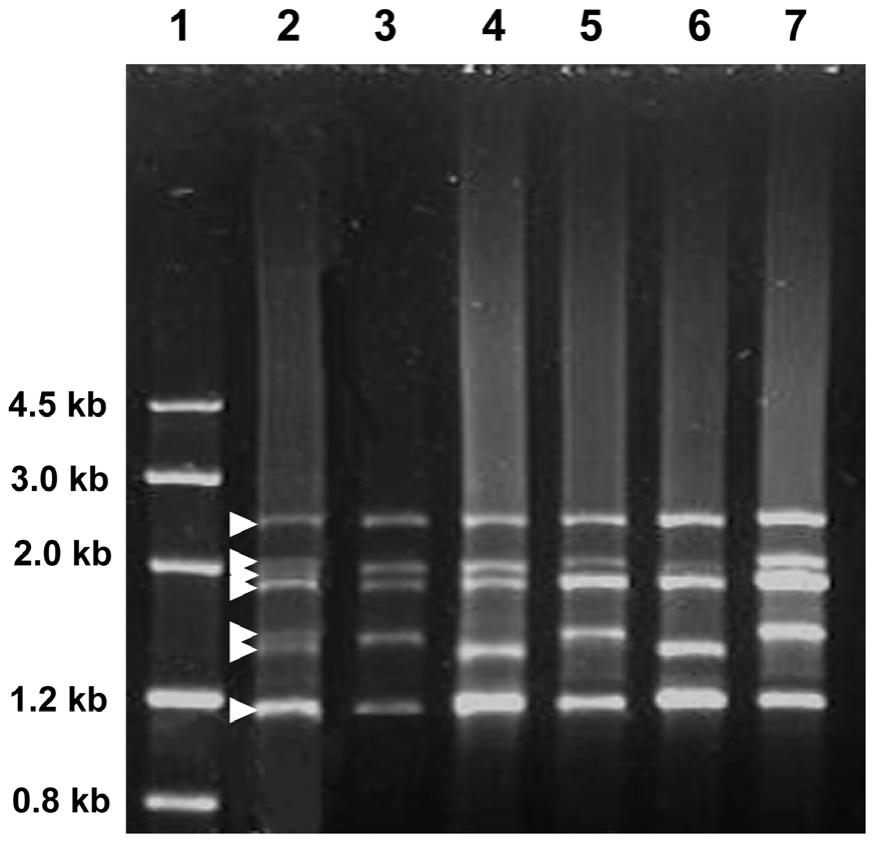
Amplification of complete ORFs coding for HMW-GSs of *Ag. intermedium*. Lane 1 is DNA marker III from TIANGEN and lane 2∼7 shows the PCR bands from *Ag. intermedium* seedlings corresponding to the seeds of lane 2∼7 in Figure 1.

In genomic PCR experiments using the primers P1 and P2, tens of DNA fragments (lanes 2∼7 in Figure 2) were generated from *Ag*. *intermedium* individual plants corresponding to the representative seeds with different HMW-GS genotypes in Figure 1. After cloning these fragments into the plasmid vector pGEM-T, at least three positive clones for each insert were sequenced. The sequencing results at both ends showed that the positive clones of the seven inserts from the lane 2 of Figure 2 included those of the inserts from the other individual plants (lanes 3∼7 in Figure 2). Subsequently, the positive clones of seven inserts, designated as p1Ai1-2.4, p1Ai2-2.1, p1Ai3-1.9, p1Ai4-1.8, p1Ai5-1.5, p1Ai6-1.4 and p1Ai7-1.2, were completely sequenced by overlapping the subclones prepared from nested deletion method. Sequence analysis using the ORF Finder program (http://www.ncbi.nlm.nih.gov/projects/gorf/) indicated that the seven genes all possessed complete HMW-GS ORFs and were deduced to represent the ORFs of the 1Ai1, 1Ai2, 1Ai3, 1Ai4, 1Ai5, 1Ai6 and 1Ai7 subunits, respectively (lane 2 in Figure 1). According to the sequencing results, the lengths of the seven ORFs were in decreasing order 2442, 2046, 1908, 1770, 1500, 1386 and 1149 bp and encoded 793, 661, 615, 569, 479, 441 and 362 amino acid residues, respectively (Table 2). Of these, 1Ai4, 1Ai5, 1Ai6 and 1Ai7 ORFs were unusually small compared to the representatives of wheat HMW-GS genes. Furthermore, blast analysis showed that 1Ai4 ORF was completely identical to that of 1Aix1 (accession number DQ304542 in GenBank), an *Ag. intermedium* HMW-GS isolated from *T. aestivum*-*Ag. intermedium* addition lines TAI-13 [10], whereas the other six differed from previously published HMW-GSs (http://blast.ncbi.nlm.nih.gov/Blast.cgi).

**Table 2:** Properties of the primary structure of the HMW-GS from *Ag. intermedium* in comparison with those of the representatives of wheat HMW-GS.

Subunit	ORF^a^	N-terminal		C-terminal		Rep. domain		Total		Renamed subunit	Gene
		Size	Cys^b^	Size	Cys^b^	Size	Cys^b^	Size	Cys^b^		
1Ai1	2442	86	3	42	1	665	0	793	4	1Aix2	*Glu-1Aix2*
1Ai2	2046	104	5	42	1	515	0	661	6	1Aiy1	*Glu-1Aiy1*
1Ai3	1908	104	5	42	1	469	1	615	7	1Aiy2	*Glu-1Aiy2*
1Ai4	1770	81	3	42	1	446	2	569	6	1Aix1	*Glu-1Aix1*
1Ai5	1500	81	3	42	1	356	0	479	4	1Aix3	*Glu-1Aix3*
1Ai6	1386	81	3	42	1	318	0	441	4	1Aix4	*Glu-1Aix4*
1Ai7	1149	105	5	42	1	215	2	362	8	1Aiy3	*Glu-1Aiy3*
1Ax2*	2445	86	3	42	1	666	0	794	4		
1Bx7	2367	81	3	42	1	645	0	768	4		
1Dx5	2544	89	3	42	1	696	1	827	5		
1Ay	1761	104	5	42	1	420	0	566	6		
1By9	2115	104	5	42	1	538	1	684	7		
1Dy10	1944	104	5	42	1	481	1	627	7		

a The nucleotide number of Open Reading Frame;

b The number of Cys.

### Expression in *E. coli* and Western Blotting Analysis of HMW-GSs

**Figure 3:**
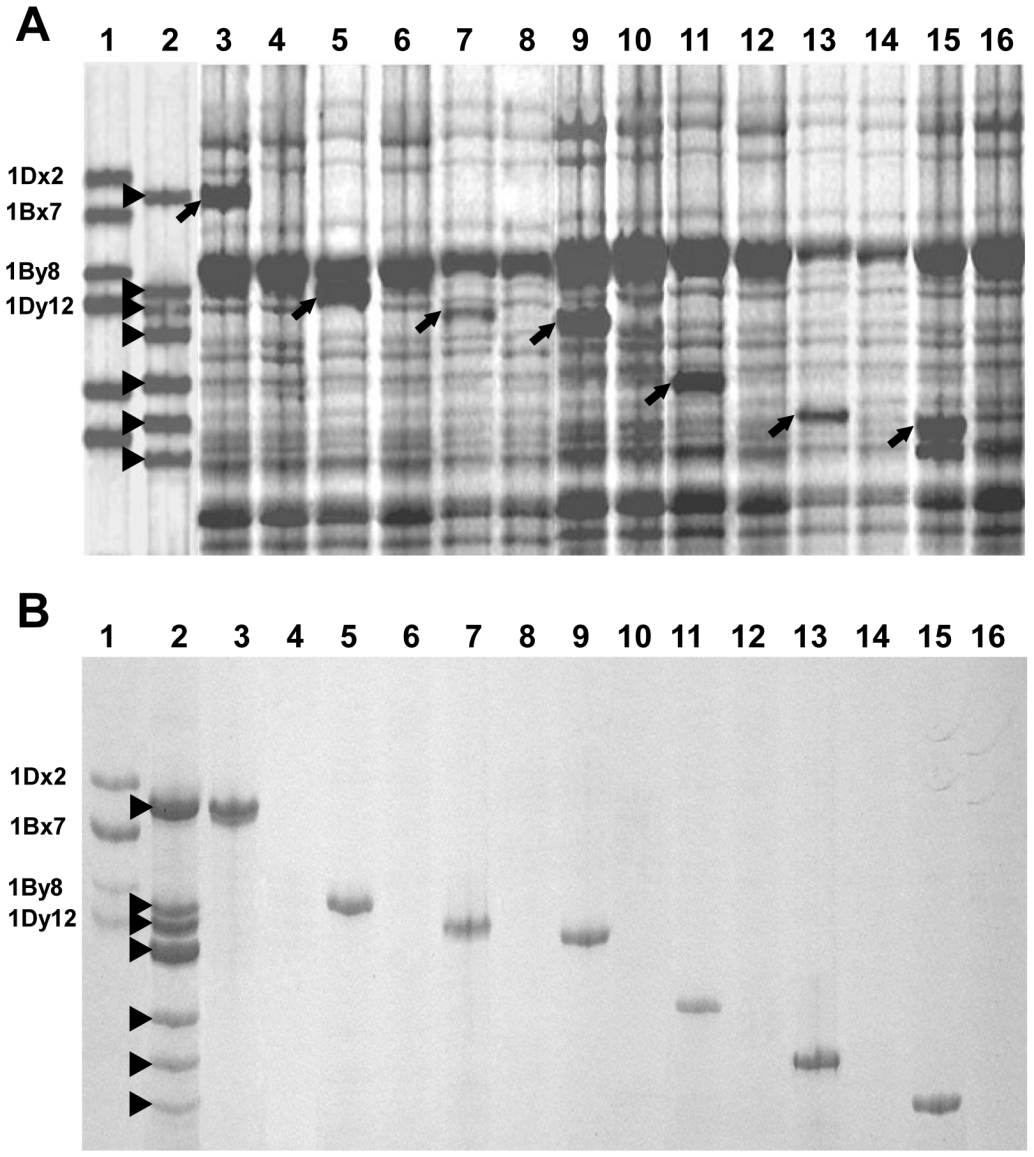
SDS-PAGE (A) and Western blotting (B) analysis of HMW-GSs from *Ag. intermedium* and bacterial expression products. Lane 1 is HMW-GSs from common wheat variety Chinese Spring, the four expressed HMW-GSs are noted on left; Lane 2 is native HMW-GS from the seed of *Ag. intermedium* same as the lane 2 in Figure 1, the seven expressed HMW-GSs are marked with triangles; Lane 3, 5, 7, 9, 11, 13 and 15 are total cell proteins from IPTG induced *E. coli* containing pET-*Glu-1Ai1*, pET-*Glu-1Ai2*, pET-*Glu-1Ai3*, pET-*Glu-1Ai4*, pET-*Glu-1Ai5* pET-*Glu-1Ai6* and pET-*Glu-1Ai7*, respectively, whereas the dextral lanes for each of them shows the total cell proteins from their bacterial cells without induction of IPTG. The seven expressed target proteins in *E. coli*, which were detected by SDS-PAGE (marked with arrows in A) and were confirmed by Western blotting (lanes 3, 5, 7, 9, 11, 13 and 15 in B), share comparable electrophoretic mobility with those native HMW-GSs from *Ag. intermedium* (lane 2).

To confirm the cloned *Ag*. *intermedium* HMW-GS genes, they were subjected to bacterial expression and Western blotting analysis. For bacterial expression, the nucleotide sequence encoding the signal peptide was removed from the cloned ORF by PCR mutagenesis. This ensured the synthesis of the mature proteins of the cloned genes, which should possess comparable electrophoretic mobility with those of the native subunits from the seeds. Seven expression constructs, pET-*Glu-1Ai1*, pET-*Glu-1Ai2*, pET-*Glu-1Ai3*, pET-*Glu-1Ai4*, pET-*Glu-1Ai5* pET-*Glu-1Ai6* and pET-*Glu-1Ai7*, were created for expressing the mature proteins of the 1Ai1, 1Ai2, 1Ai3, 1Ai4, 1Ai5, 1Ai6 and 1Ai7 subunits in *E. coli* cells, respectively. In SDS-PAGE analysis showed that the electrophoretic mobility of the seven proteins directed by the seven expression constructs above, (lanes 3, 5, 7, 9, 11, 13 and 15 in Figure 3A shown by triangles) were in accordance with their native subunits extracted from the seed (lane 2 in Figure 3A), respectively. Furthermore, the seven bacterially expressed subunits were exhibited strong reactions to the polyclonal antibody specific for HMW-GSs in Western blotting (Figure 3B).

### Characteristics of the Amino Acid Sequences Deduced from *Ag. intermedium* HMW-GS Genes

Analysis of amino acid sequences derived from the cloned seven genes showed that HMW-GSs in *Ag*. *intermedium* possessed the same primary structure as known HMW-GSs, with the conserved signal peptide (which was excised from the mature proteins), N- and C-terminal domains, and the central repetitive region composed of tri-, hexa- and nona-peptides motifs [3]. Some of their properties, in comparison to those of the representatives of wheat HMW-GSs, are summarized in Table 2. There were 3 or 5 cysteine residues in their conserved N-terminal region and 1 cysteine residue in their conserved C-terminal region, and 1Ai4 and 1Ai7 had at least one extra cysteine residue in their repetitive regions compared to the representatives of wheat HMW-GSs (Table 2). Judging from the size of conserved N-terminal domains (especially the number of the cysteine residues), 1Ai1, 1Ai4, 1Ai5 and 1Ai6 belonged to x-type, whereas 1Ai2, 1Ai3 and 1Ai7 fell into y-type. The ORF of 1Ai4 was completely identical to *Glu-1Aix1* (DQ304542), an *Ag*. *intermedium* HMW-GS gene isolated previously from the *T. aestivum*-*Ag*. *intermedium* addition lines TAI-13, so it still follows the previous name [10]. Here we designated the genes of 1Ai1, 1Ai2, 1Ai3, 1Ai5, 1Ai6 and 1Ai7 as *Glu-1Aix2*, *Glu*-*1Aiy1*, *Glu*-*1Aiy2*, *Glu*-*1Aix3*, *Glu*-*1Aix4* and *Glu*-*1Aiy3*, respectively, according to their size and attributive types. Accordingly, the HMW-GSs 1Ai1∼7 in Figure 1 were renamed 1Aix2, 1Aiy1, 1Aiy2, 1Aix1, 1Aix3, 1Aix4 and 1Aiy3, respectively (Table 2). The six unique HMW-GS genes cloned in this study, *Glu-1Aix2*, *Glu*-*1Aiy1*, *Glu*-*1Aiy2*, *Glu*-*1Aix3*, *Glu*-*1Aix4* and *Glu*-*1Aiy3* have been submitted to GenBank with accession numbers, EF105400, EF105401, EF105402, EF105403, EF105404 and EF105405, respectively.

**Figure 4:**
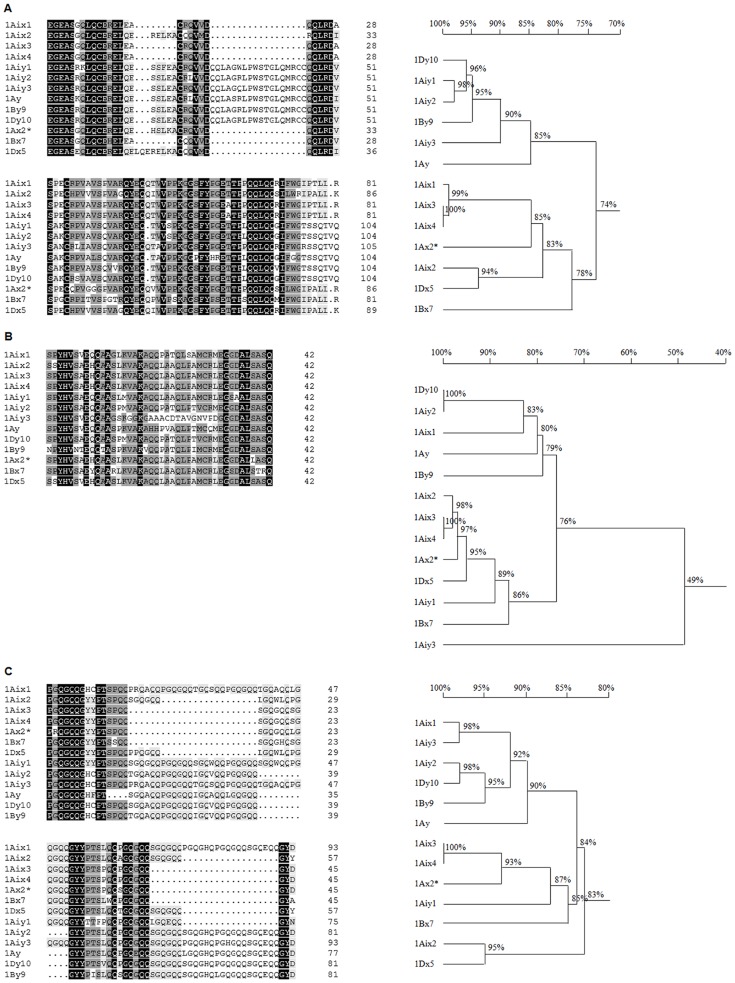
Alignments and clustering analyses based on N-terminal (A), C-terminal (B) and the last 93 residues in the repetitive region (C) of the HMW-GSs from *Ag. intermedium* and several representative HMW-GSs from *Triticum* genus. Noticeably, the subunit 1Aix1 possesses a N-terminal clustered to x-type subunits (A) and a C-terminal and last part of repetitive region clustered to y-type subunits (B and C). Conversely, the subunit 1Aiy1 possesses a N-terminal more similar to y-type subunits (A) and a C-terminal and last part of repetitive region more similar to x-type subunits (B and C). The default parameters were used for full alignment and clustering analysis of sequences by aid of DNAMAN version 5. 2. 2.

Except for 1Aiy1 and 1Aix1, the classificatory affiliations of the other subunits were confirmed by alignment and clustering analysis based on the conserved N- and C-terminal domains (Figure 4A and 4B). In the case of 1Aiy1, its N-terminal domain displayed higher similarities to those of y-type subunits, whereas a reverse pattern was found in its C-terminal domain (designated as yx-type). Conversely, 1Aix1 comprised the N-terminal of x-type and C-terminal of y-type (designated as xy-type), just as identified previously [10]. Considering that the sizes of the C-terminal amino acid sequences are too short to be used reliably in alignment and clustering analysis (Figure 4B), the near C-terminal portion of the repetitive domain of the two hybrid subunits were compared with those of the representatives of HMW-GSs (Figure 4C). The alignment analysis showed the near C-terminal portion of the repetitive domain of 1Aiy1 displayed higher similarities to those of x-type subunits than y-type subunits, whereas that of 1Aix1 showed higher similarities to those of y-type subunits than x-type subunits (left panel in Figure 4C). Consequently, in the cladogram developed from near C-terminal domain sequences, 1Aiy1 clustered with x-type subunits whereas 1Aix1 fell into the group of y-type subunits (right panel in Figure 4C), which is in line with the result from their C-terminal domain sequences. Moreover, the nonapeptide GHCPTSPQQ, which has not been found in any of the previously reported x-type subunits but existed in near C-terminal portion of the repetitive domain of the majority of the y-type subunit characterized so far, was present in the repetitive domain of 1Aix1 (left panel in Figure 4C). Conversely, 1Aiy1 lacks GHCPTSPQQ-like nonapeptide in its repetitive domain. These results further validated that both subunits belong to the hybrid types.

Here the native 1Aiy1 subunit also was subjected to MALDI-TOF-MS analysis which is effective in gaining structural information of the HMW-GSs directly isolated from seeds [26], [27]. By repeated MALDI-TOF-MS experiments, the molecular mass of sixteen peptides from 1Aiy1 could be reliably determined (Table 3), and their molecular mass values matched closely with the calculated values (columns 1 and 2 in Table 3). Together, the sixteen peptides covered the complete portion of the amino acid sequence (minus the signal peptide) deduced from *Glu*-*1Aiy1* (column 3 in Table 3). Consequently, the peptide mass fingerprint of native 1Aiy1 indicated that the deduced amino acid sequence from *Glu*-*1Aiy1* was accurate and really to be a true representation of the native subunit.

**Table 3:** MALDI-TOF-MS analysis of peptide mass fingerprint of native 1Aiy1 subunit.

Measured mass[M+H]^+^	Calculated mass[M+H]^+^	Location	Missed cleavge	Peptide sequence predicted using the bioinformatic program Peptide Mass (with trypsin digestion)
				
1406.118	1405.685	1–12	2	EGEASRKLQCER
1298.749	1298.568	13–23	0	ELQESSFEACR
2313.545	2313.18	24–43	0	QVVDQQLAGWLPWSTGLQMR
1250.654	1250.598	44–54	1	CCQQLRDVSAK
1186.176	1185.652	55–65	0	CRPVAVSQVAR
1178.789	1178.605	66–75	0	QYEQTVVSPK
4251.455	4250.046	76–114	0	GGSFYPGETTPLQQLQQGIFWGTSSQTVQGYYPSVTSPR
2287.455	2288.069	115–136	0	QGSYYPGQASPQQPGQGQQPGK
4771.798	4770.21	137–179	0	WQEPGQGQQGYYPTSLQQPGQGQQIGQGQQGYYPTSPQHPGQR
6854.134	6852.202	180–240	0	QQPVQGQQIGQGQQPEQGQQPGQWQQGYYPTSPQQPGQGQQPGQWQEPGQGQQGYYLIFSR
10014.945	10016.624	241–332	0	SWGQGQQIGQGQHPGQGHQGYYPFSPQPPGQWAQPGQGQQPGQLQQGYYPFSPQQPGQGQHGYYPTFSAAPGQGYYPTFPHQPGQGQQGQR
13765.658	13762.182	333–457	0	QHPGQGQQGYYPTFSQQPGQWQQSGQGHQGYYPTSPQQSGQGQQGYYPTFSAAPGQGYYPTFPHQSGQGQQPGQGQQPGQGYYPTFPQLPGQGQQGYYPTYPQQSGQGQQPGQWQQSGQWQQPGR
5481.774	5479.452	458–507	0	GQQSGQWQQPGQGQQSGQWQQPGQGQQGYYPTSPQQSGQGQQGYYPTSPR
14046.246	14045.376	508–637	0	QPGQGYYPTFPHQSGQGQQPGQGQQPGQGYYPTFPQPPGQGQQGYYPTSPQQSGQGQQPGQGQQSGQWQQPGQGQQSGQWQQPGQGQQGYYTTFPQQPGQGQQLGQEQQGYNSPYHVSAEQQAASLMVAK
1400.645	1400.713	638–650	0	AQQLAAQLPAMCR
1035.351	1035.516	651–661	0	LEGSAALSASQ

Peptide mass is available at http://www.expasy.org/tools/peptide-mass.html.

### Phylogenetic Analysis

**Figure 5:**
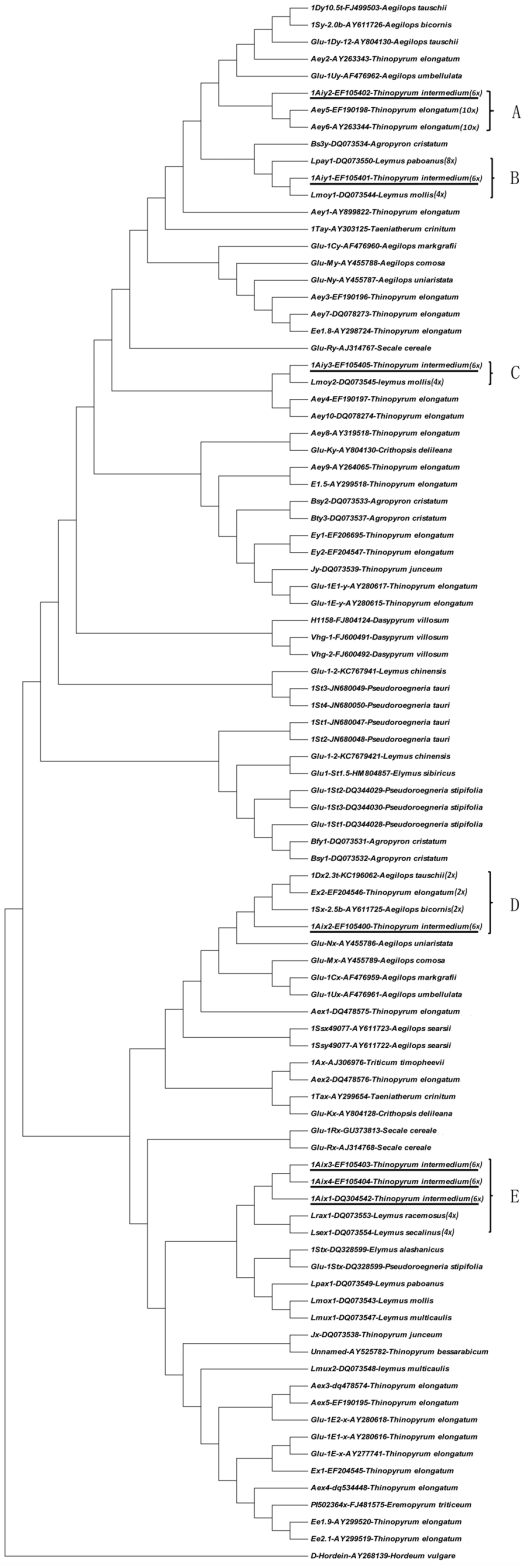
Phylogenetic tree of *Thinopyrum intermedium* ( = *Ag. intermedium*) and some representative HMW-GSs from *Triticeae*. This phylogenetic tree was constructed with Maximum Likelihood Estimation method based on the nucleotide sequences encoding signal peptide and N-terminal conserved region of HMW-GSs plus the next three repeat units, one dodecapeptide, one undecapeptide and one hexapeptide repeat. D-hordein from barley was used as outgroup. The species names of HMW-GS genes in this figure are consistent with their accession names in GenBank, so here we replaced *Agropyron intermedium* with *Thinopyrum intermedium*.

Along with the development of molecular biology, the study of evolutionary biology demands for more elaborate and comprehensive data. Comparative genetics demonstrated that chromosome group 1 is very well conserved in *Triticeae* [28]. Moreover, the gene-rich region in *Glu-1* loci of chromosome group 1 indicated very good microcolinearity [29]. This region is therefore a good target to study evolution events at the molecular level. Recently, the genomic evolution of common wheat was surveyed through the comparative genomics of *Glu-1* loci [29], [30]. In addition, a lot of research showed that the coding sequences for HMW-GSs from *Glu-1* loci are phylogenetically informative and even were used to estimate the divergence time of genomes [9], [31], [32], [33]. Most importantly, a large amount of information about HMW-GS genes from *Triticeae* is already available from other studies. Here we created a phylogenetic tree (Figure 5) based on the conserved sequences encoding the signal peptide and N-terminal region of *Ag. intermedium* HMW-GSs and the representative HMW-GSs from the major genomes in the tribe *Triticeae*, such as A, B, C, D, E, G, H, K, M, N, P, R, S, St, T, U, V etc [34]. In the phylogenetic tree, regardless of x- or y-types, the HMW-GSs from *Ag. intermedium* have closest distance with those from *Leymus* genus and *Thinopyrum elongatum* ( = *Agropyron elongatum* = *Elytrigia elongata*), (subgroups A, B, C, D and E in Figure 5). Only subunit 1Aix2 has also close relationship with those from *Aegilops* with S^b^ or D genome (subgroup D in Figure 5). In contrast, none of *Ag. intermedium* HMW-GS was classified into close branch with those from *Thinopyrum bessaracicum* (E^b^), *Thinopyrum junceum* (J) and *Pseudoroegneria* species (having St genome).

## Discussion

The HMW-GSs and their coding genes from an *Ag*. *intermedium* line were comprehensively analyzed in this study. SDS-PAGE and Western blotting analysis of seed proteins indicated that the *Ag*. *intermedium* line aggregately expressed seven unique HMW-GSs. The putative genes encoding the seven subunits were isolated from genomic DNA with PCR method. The further experiments on bacterial expression and Western blotting analysis confirmed that the seven genes were active, leading to the synthesis of HMW-GSs in the seeds of the *Ag*. *intermedium* line. Amino acid sequence comparisons showed that the seven HMW-GSs from *Ag*. *intermedium* possessed high similarity to those from wheat (Figure 4), indicating that the *Glu-1* loci of *Ag*. *intermedium* were closely related to those of wheat. However, the HMW-GSs 1Aix1, 1Aix3, 1Aix4 and 1Aiy3 subunits from *Ag*. *intermedium* are substantially smaller than those from Chinese Spring. Of them, 1Aiy3 probably is one of the smallest HMW-GSs published so far. Judging from the sizes of their HMW-GS genes (approximately 1.15∼2.44 Kb), the degree of diversity among the three genomes (E_1_E_2_X) of *Ag*. *intermedium* is considerably higher than that of wheat, from which the sizes of HMW-GS genes are about 2.0∼2.5 Kb. In addition, there is large variation of genotypes even in a single *Ag*. *intermedium* line (Figure 1 and 2) because *Ag*. *intermedium* is in general cross-pollinated. Thus, *Ag*. *intermedium* may supply abundant variation of HMW-GSs for the quality improvement of wheat. Especially for these unusually small subunits, it may be interesting to research their effect on end-use quality of wheat.

It is well known that the HMW-GSs from not only common wheat but also its relatives are encoded by the *Glu-1* locus containing the *Glu-1-1* (x-type) and *Glu-1-2* (y-type) genes. For common wheat with three genomes (A, B and D), there generally are 3∼5 HMW-GSs for each line due to *Glu-1A-2* (y-type) gene is inactivated in most if not all cases (reviewed in [7]). Similarly, there are 3∼5 HMW-GSs in most of seeds from the *Ag*. *intermedium* line (Figure 1). This result, together with previous findings, suggested that there was one genetic locus of *Glu-1* on each chromosome 1 from three genomes of *Ag*. *intermedium* [16], [35], [36]. However, seven HMW-GSs were observed in one of the seeds from the *Ag*. *intermedium* line used in this study (lane 2 in Figure 1). We speculated that there was at least one heterozygous *Glu-1* considering that *Ag*. *intermedium* was a naturally cross pollinated species. Based on the alignment and clustering analysis of N- and C-terminals as well as the portions of repetitive region near C-terminals (Figure 4), the identity of *Glu-1Aix3* and *Glu-1Aix4* was 100%, which suggested that the two genes likely were alleles of the heterozygous *Glu-1*.

**Figure 6:**
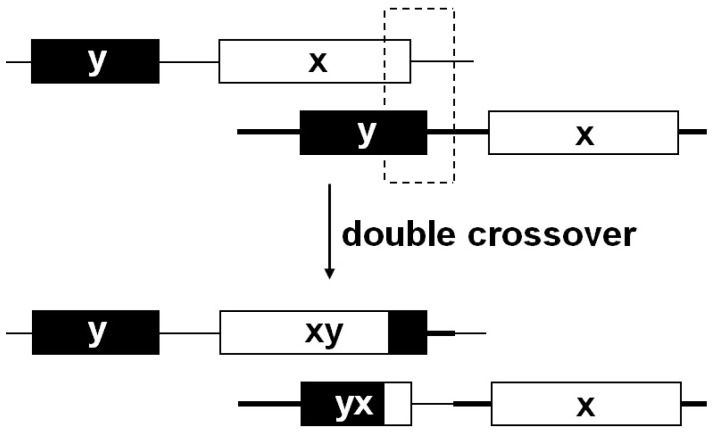
Illustration for the developmental mechanism of two hybrid HMW-GSs based on unequal double crossover hypothesis. The broken line box indicates the double crossover region. The xy and yx represent the hybrid subunit with 5′region of x-type and 3′region of y-type and the hybrid subunit with 5′region of y-type and 3′region of x-type, respectively.

A hybrid HMW-GS (GenBank accession No. AY611724) was isolated from *Ae*. *searsii*, and it was hypothesized that unequal crossover between the two *Glu-1* loci located on the sister chromatids of the homologous chromosomes during meiosis might have led to the formation of the novel hybrid ORF [9]. Following this mechanism underlying the evolution of the subunit, this would generate two hypothetical recombinant *Glu-1* loci, which belong to xy-type and yx-type, respectively (Figure 6). In our study, two hybrid subunits, xy-type subunit 1Aix1 and yx-type subunit 1Aiy1, were identified from one *Ag*. *intermedium* line, which strongly supported the hypothetic mechanism above. In addition to the configuration shift of the subunits, the size change of the subunits might be resulted from the unequal crossover (Figure 6).

In previous literature on HMW-GSs, quite a few research results showed that cysteine residues are involved in the formation of inter- or intra-molecular disulphide bonds [6], [7], [8] and in addition there is little doubt that this network of GMP is stabilized by inter-chain disulphide bonds [8]. Most importantly, it has been shown that the 1Dx5 subunit with an additional cysteine residue in its repetitive region exerts a positive influence on bread-making quality of flour [37], [38]. Considering these distinctive characteristics on the location and/or number of cysteine residue in 1Aix1, 1Aiy1 and 1Aiy3 (Table 2) from *Ag*. *intermedium*, it is quite necessary to identify their effect on the quality of flour.

During the recent decades, cytogenetic research showed that *Ag. intermedium* is an autoallo-hexaploid species designated with genomes E1E1E2E2XX, where the E1, E2 and X genomes are known to be related to the E^e^ genome (similar to J genome [34]) of diploid *Th. elongatum*, the E^b^ genome of *Th. bessarabicum* and the St genome of *Ps. stipifolia*, respectively [11], [25], [39], [40]. However, our phylogenetic analysis based on HMW-GS genes revealed the two of the three species (*Th. bessarabicum* and *Ps. stipifolia*) are not close to *Ag. intermedium* in term of evolution (Figure 5). Considering *Ag. intermedium* is in general cross pollinated and that there is a wide variability within a single accession, the *Ag. intermedium* accession in this study probably is different from those in the cytogenetics studies. Thus, our data only displayed the putative origin of the specific *Ag. intermedium* accession in our study. In our phylogenetic analysis, another remarkable point is that some *Aegilops* species with D (*Ae. tauschii*) or S^b^ (*Ae. bicornis*) genome probably are involved in the origin of *Ag. intermedium* (subgroup D of Figure 5). In addition, recent research showed that all of the *Ag*. *intermedium*-wheat addition lines with alien HMW-GS lost the activity of Glu-D1 [41], so at least one donor of *Ag. intermedium* is exchangeable with wheat D group chromosomes, i.e. a wheat D group-like genome probably is involved in the origin of *Ag*. *intermedium*. Furthermore, *Ag*. *intermedium* is known commonly as “*intermedium* wheatgrass” because of the similarity of their seed “heads or ears” to wheat. Most important is their high crossability with wheat, which is very helpful for germplasm innovation. As such, it is inferred that the common or similar genomic donors of wheat and *Ag. intermedium* probably contribute to their similar phenotypes and high crossability. For *Leymus*, with two basic genomes Ns and Xm [34], some of its species are the most likely direct donors of the *Ag. intermedium* based on the phylogenetic tree (subgroups B, C and E of Figure 5). In all, our phylogenetic analysis expanded the understanding on the origin of *Ag. intermedium*. However, more evidence is necessary to confirm our phylogenetic result using cytogenetics and/or comparative genomics approaches.
